# Crystal structure of di-μ-chlorido-bis­[chlorido­bis­(1,2-dimethyl-5-nitro-1*H*-imidazole-κ*N* ^3^)copper(II)] acetonitrile disolvate

**DOI:** 10.1107/S2056989016015413

**Published:** 2016-10-25

**Authors:** Patrick J. Quinlivan, Rita K. Upmacis

**Affiliations:** a Department of Chemistry, Columbia University, New York, NY 10027, USA; b The Haskins Laboratories, Department of Chemistry and Physical Sciences, Pace University, New York, NY 10038, USA

**Keywords:** crystal structure, copper(II), chloride-bridged dimer, dimetridazole, dimet

## Abstract

1,2-Dimethyl-5-nitro­imidazole (dimetridazole, dimet) is a compound that belongs to a class of nitro­imidazole drugs that are effective at inhibiting the activity of certain parasites and bacteria. However, there are few reports that describe structures of compounds that feature metals complexed by dimet. Therefore, we report here that dimet reacts with CuCl_2_·H_2_O to yield a chloride-bridged copper(II) dimer, [Cu_2_Cl_4_(C_5_H_7_N_3_O_2_)_4_] or [Cu(μ-Cl)Cl(dimet)_2_]_2_. In this mol­ecule, the Cu^II^ ions are coordinated in an approximately trigonal–bipyramidal manner, and the mol­ecule lies across an inversion center. The dihedral angle between the imidazole rings in the asymmetric unit is 4.28 (7)°. Compared to metronidazole, dimetridazole lacks the hy­droxy­ethyl group, and thus cannot form inter­molecular O⋯H hydrogen-bonding inter­actions. Instead, [Cu(μ-Cl)Cl(dimet)_2_]_2_ exhibits weak inter­molecular inter­actions between the hydrogen atoms of C—H groups and (i) oxygen in the nitro groups, and (ii) the terminal and bridging chloride ligands. The unit cell contains four disordered aceto­nitrile mol­ecules. These were modeled as providing a diffuse contribution to the overall scattering by SQUEEZE [Spek (2015 ▸). *Acta Cryst.* C**71**, 9–18], which identified two voids, each with a volume of 163 Å^3^ and a count of 46 electrons, indicative of a total of four aceto­nitrile mol­ecules. These aceto­nitrile mol­ecules are included in the chemical formula to give the expected calculated density and *F*(000).

## Abstract 2

1,2-Dimethyl-5-nitro­imidazole (dimetridazole, dimet) reacts with copper(II) chloride to give dinuclear [Cu(dimet)_2_(*μ*-Cl)Cl]_2_, in which each copper moiety is coordinated to two dimet ligands in a *trans* arrangement.

## Chemical context   

**Figure 1:**
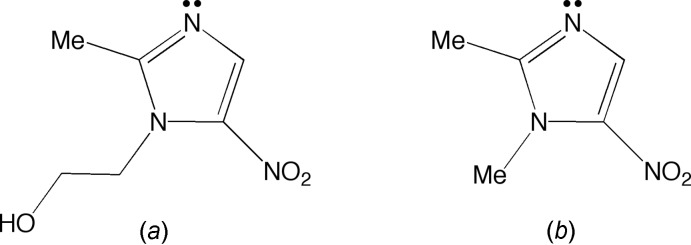
A comparison of the structures of (*a*) metronidazole (MET) and (*b*) dimetridazole (dimet).

1,2-Dimethyl-5-nitro­imidazole, also known as dimetridazole (dimet), is structurally related to metronidazole [2-(2-methyl-5-nitro-1*H*-imidazol-1-yl)ethanol, MET]. Thus, both compounds contain a 2-methyl-5-nitro­imidazole core and are only differentiated according to whether one of the nitro­gen atoms possesses a methyl substituent (as in dimet) or a hy­droxy­ethyl substituent (as in MET), as illustrated in Fig. 1 ▸. Both MET and dimet are used to treat microbial infections, but dimet has specifically been used in animals for the treatment of, for instance, bovine trichomoniasis (McLoughlin, 1968 ▸), giardiasis in birds (Panigrahy *et al.*, 1978 ▸) and swine dysentery (Messier *et al.*, 1990 ▸). In order to control outbreaks of infection, a previous common practice was to incorporate dimet as a feed additive given, for example, to poultry and pigs (Buizer & Severijnen, 1975 ▸). However, concerns about the mutagenic properties displayed by this class of drug (Voogd *et al.*, 1974 ▸), and the fact that trace amounts can be detected in certain animal products intended for human consumption (Arias *et al.*, 2016 ▸), have led to a discontinuation of this practice (*EC bans use of dimetridazole in food animals*, 1995 ▸). Reports of structures of metal compounds involving the coordination by dimetridazole are scarce. Herein, we describe the structure of the copper compound [Cu(μ-Cl)Cl(dimet)_2_]_2_, which is obtained by the reaction of dimet with CuCl_2_·H_2_O (see Scheme).
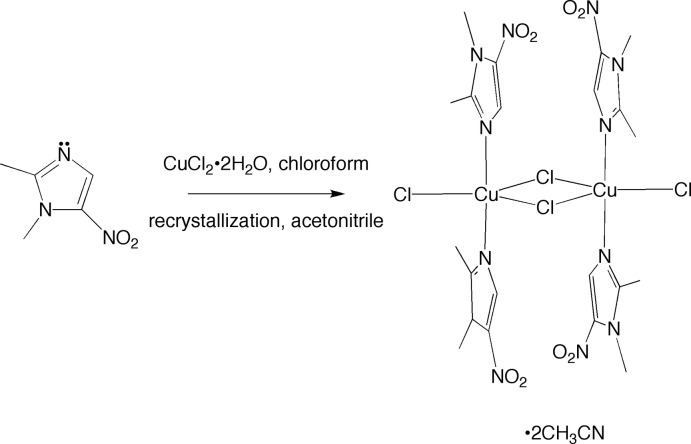


## Structural commentary   

**Figure 2:**
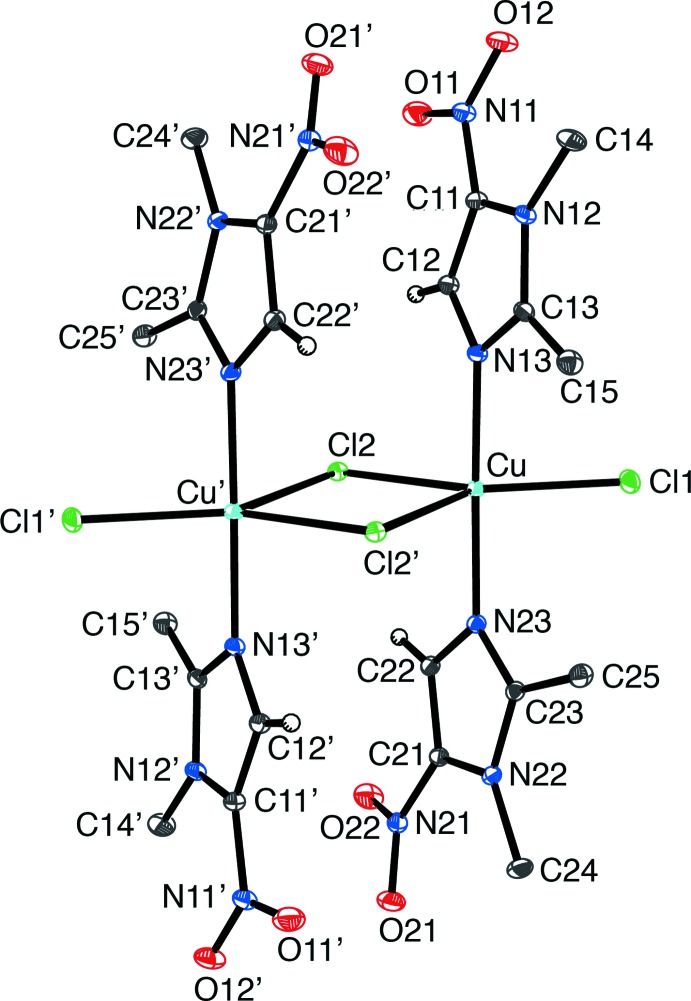
The mol­ecular structure of [Cu(μ-Cl)Cl(dimet)_2_]_2_, with displacement ellipsoids depicted at the 30% probability level. H atoms associated with methyl groups are not shown [symmetry code (’): −*x*, −*y* + 1, −*z* + 1].

**Figure 3:**
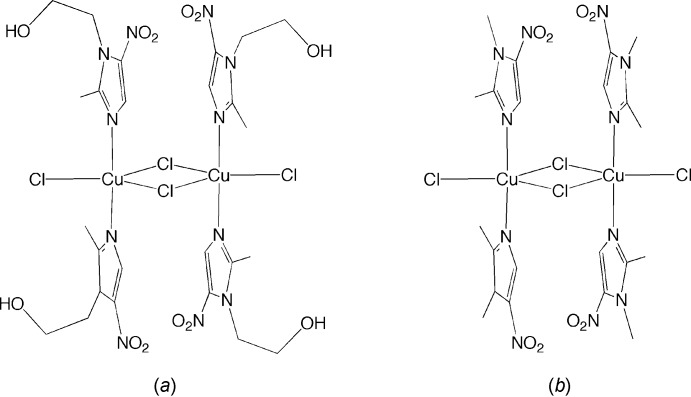
A comparison of the structures of the dinuclear Cu complexes which are derived from (*a*) metronidazole (MET) and (*b*) dimetridazole (dimet).

Crystals of composition [Cu(μ-Cl)Cl(dimet)_2_]_2_ were obtained by addition of dimet to CuCl_2_·2H_2_O in chloro­form, followed by recrystallization of the blue precipitate from aceto­nitrile. The molecular structure, as illustrated in Fig. 2 ▸, shows a centrosymmetric chlorido-bridged dimer. The coordination geometry around each copper atom is a slightly distorted trigonal–bipyramidal with two axial dimet ligands, and three chlorine ligands in the equatorial plane, two of which bridge to the adjacent copper. This structure is analogous to a previously reported copper(II) dimer containing MET, instead of dimet, [Cu(MET)_2_(μ–Cl)Cl]_2_ (Barba-Behrens *et al.*, 1991 ▸), and a comparison of the two structures is shown in Fig. 3 ▸. Other recent examples of metal compounds containing MET include: Cu(MET)_2_Cl_2_, [Ag(MET)_2_](BF_4_), and [H(MET)][AuCl_4_] (Palmer *et al.*, 2015 ▸; Palmer & Upmacis, 2015 ▸; Quinlivan *et al.*, 2015 ▸).

Examination of the structure of the [Cu(μ-Cl)Cl(dimet)_2_]_2_ complex demonstrates that, inter­estingly, the chlorine atoms bridge in an asymmetric manner, with Cu—Cl_bridge_ bond lengths of 2.3811 (3) and 2.6024 (3) Å, both of which are longer than the terminal Cu—Cl bond length of 2.2822 (3) Å. Of note, these features are also observed for the MET analog, [Cu(MET)_2_(μ–Cl)Cl]_2_, which possesses bridging Cu—Cl distances of 2.418 (1) and 2.619 (1) Å, and a terminal bond length of 2.297 (2) Å (Barba-Behrens *et al.*, 1991 ▸). Furthermore, the Cu—N bond lengths [2.0009 (10) and 1.9914 (9) Å] are also similar to the Cu—N bond lengths reported for the MET analog [2.002 (4) and 1.993 (4) Å]. In terms of the bond angles, the N—Cu—Cl_term_ and N—Cu—Cl_bridge_ angles are all close to 90° [ranging from 88.95 (3)° for N13—Cu—Cl1 to 91.85 (3)° for N23—Cu—Cl2], with the exception of N23—Cu—Cl2_bridge_ which is 85.42 (3) Å.

## Supra­molecular features   

**Figure 4:**
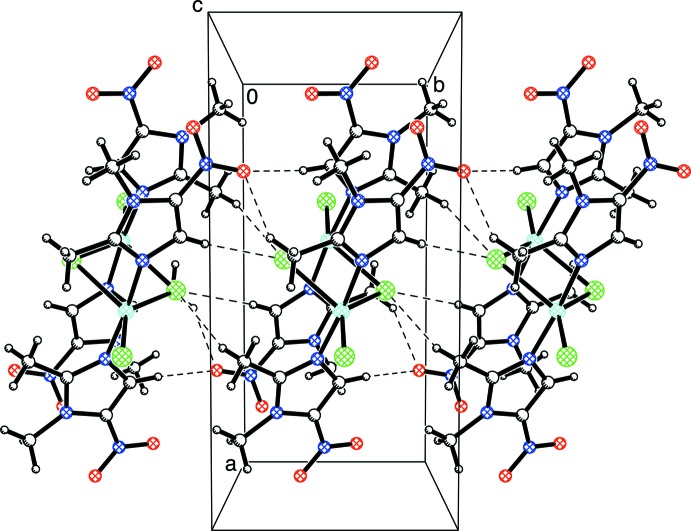
Weak inter­molecular hydrogen-bonding inter­actions (shown as dashed lines) for [Cu(μ-Cl)Cl(dimet)_2_]_2_.

The crystal structure displays a number of weak inter­molecular inter­actions between hydrogen atoms of CH groups and the more electronegative atoms on adjacent mol­ecules, such as the oxygen atoms in the nitro groups of the dimet ligand and also the terminal and bridging chlorine atoms (see Table 1 ▸ and Fig. 4 ▸). In this regard, one of the oxygen atoms of the nitro group participates in inter­molecular hydrogen-bonding inter­actions with CH_3_ and CH groups of an adjacent mol­ecule. For reference, inter­molecular and intra­molecular C—H⋯O hydrogen bonds involving an O atom from a nitro group (or other O-containing groups) have been reported (Desiraju, 1991 ▸; Sharma & Desiraju, 1994 ▸; Forlani, 2009 ▸). As an illustration, inter­molecular C—H⋯O inter­actions (involving C—H motifs from an NMe_2_ substituent and the O atoms of a nitro group) are reported at 2.71 (3) Å, with C⋯O distances of 3.658 (4) and 3.725 (4) Å (Sharma & Desiraju, 1994 ▸). The results of our structure analysis are also comparable to the average values that have been reported for hydrogen-bonding inter­actions of (N,C)C*sp* ^2^—H (2.48 and 3.47 Å) and C*sp* ^3^—CH_3_ (2.63 and 3.61 Å) groups with a water O atom (Steiner, 2002 ▸). For comparison, intra­molecular N—H⋯O inter­actions to an O atom of a nitro substituent form shorter contacts, *e.g.* 1.927 (15) Å for *N*-(2-nitro­phen­yl)benzamide (Saeed & Simpson, 2009 ▸) and 2.11 Å for 2-iodo-*N*-(2-nitro­phen­yl)benzamide (Wardell *et al.*, 2005 ▸), which is in accord with the reports that C—H⋯O bonds are weaker than N—H⋯O bonds (Desiraju, 1991 ▸).

The bridging chlorine atoms also form weak inter­molecular inter­actions with CH_3_ and CH groups of an adjacent mol­ecule. In addition, the terminal chlorine atom participates in a hydrogen-bonding inter­action with a CH_3_ group of an adjacent mol­ecule.

While C—H⋯O inter­actions are widely accepted (Desiraju, 1991 ▸), C—H⋯Cl inter­actions are considered more controversial, but a survey of the literature reveals that they also represent a common phenomenon (Aakeröy *et al.*, 1999 ▸). For example, hydrogen-bonding inter­actions of *sp* ^2^ (N,C)C—H with Cl^−^ are reported at 2.64 Å (Kovacs & Varga, 2006 ▸). However, when Cl is bonded to a metal, the average C—H⋯Cl—*M* hydrogen-bonding distance is 2.974 Å (Thallapally & Nangia, 2001 ▸).

Fig. 4 ▸ illustrates some of these inter­molecular inter­actions. An important difference between this structure and the MET analog is that the dimet compound lacks the hy­droxy­ethyl group, which is involved in classical inter­molecular hydrogen-bonding inter­actions for the MET derivative (Barba-Behrens *et al.*, 1991 ▸).

## Database survey   

There is only one structurally characterized metal compound containing dimet listed in the Cambridge Database (CSD Version 5.37; Groom *et al.*, 2016 ▸), namely, a mononuclear cobalt complex, [Co(dimet)_2_Cl_2_], in which the cobalt(II) atom is surrounded by two dimet and two chlorido ligands in a distorted tetra­hedron (Rosu *et al.*, 1997 ▸; Idešicová *et al.*, 2012 ▸). The Co—N distances are reported to be 2.228 (2) and 2.035 (4) Å (Rosu *et al.*, 1997 ▸).

## Synthesis and crystallization   

CuCl_2_·H_2_O (3 mg, 0.018 mmol) was added to a solution of dimet (6 mg, 0.043 mmol) in chloro­form (0.7 mL), resulting in the precipitation of a blue solid over the course of 1 h at room temperature. The blue solid was isolated by deca­ntation and crystals of [Cu(μ-Cl)Cl(dimet)_2_]_2_, suitable for X-ray diffraction, were obtained by slow evaporation from a solution in aceto­nitrile.

## Refinement   

Crystal data, data collection and structure refinement details are summarized in Table 2 ▸. Hydrogen atoms on carbon were placed in calculated positions (C—H = 0.95–1.00 Å) and included as riding contributions with isotropic displacement parameters *U*_iso_(H) = 1.2*U*_eq_(C*sp* ^2^) or 1.5*U*_eq_(C*sp* ^3^). The unit cell contains four disordered aceto­nitrile mol­ecules. In view of the disorder, the aceto­nitrile mol­ecules were modeled as providing a diffuse contribution to the overall scattering by SQUEEZE (Spek, 2015 ▸), which identified two voids, each with a volume of 163 Å^3^ and a count of 46 electrons, indicative of a total of four aceto­nitrile mol­ecules.

## Supplementary Material

Crystal structure: contains datablock(s) I. DOI: 10.1107/S2056989016015413/lh5820sup1.cif

Structure factors: contains datablock(s) I. DOI: 10.1107/S2056989016015413/lh5820Isup2.hkl

CCDC reference: 1507712

Additional supporting information: crystallographic information; 3D view; checkCIF report

## Figures and Tables

**Table 1:** Hydrogen-bond geometry (Å, °)

*D*—H⋯*A*	*D*—H	H⋯*A*	*D*⋯*A*	*D*—H⋯*A*
C25—H25*C*⋯O22^i^	0.98	2.39	3.2804 (17)	150
C15—H15*B*⋯Cl2^i^	0.98	2.73	3.6737 (13)	163
C12—H12*A*⋯O22^ii^	0.95	2.51	3.4029 (16)	156
C22—H22*A*⋯Cl2^ii^	0.95	2.75	3.6828 (12)	167
C24—H24*C*⋯Cl1^iii^	0.98	2.84	3.7555 (13)	156

Symmetry codes: (i) 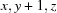; (ii) 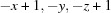; (iii) 
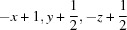
.

**Table 2:** Experimental details

Crystal data	
Chemical formula	[Cu_2_Cl_4_(C_5_H_7_N_3_O_2_)_4_]·2C_2_H_3_N
*M*_r_	915.53
Crystal system, space group	Monoclinic, *P*2_1_/*c*
Temperature (K)	130
*a*, *b*, *c* (Å)	13.9545 (8), 6.7004 (4), 19.5031 (11)
β (°)	96.424 (1)
*V* (Å^3^)	1812.10 (18)
*Z*	2
Radiation type	Mo *K*α
μ (mm^−1^)	1.54
Crystal size (mm)	0.35 × 0.17 × 0.10

Data collection	
Diffractometer	Bruker APEXII CCD
Absorption correction	Multi-scan (*SADABS*; Bruker, 2010 ▸)
*T*_min_, *T*_max_	0.637, 0.746
No. of measured, independent and observed [*I* > 2σ(*I*)] reflections	28822, 5564, 5034
*R*_int_	0.033
(sin θ/λ)_max_ (Å^−1^)	0.716

Refinement	
*R*[*F* ^2^ > 2σ(*F* ^2^)], *wR*(*F* ^2^), *S*	0.024, 0.071, 1.26
No. of reflections	5564
No. of parameters	212
H-atom treatment	H-atom parameters constrained
Δρ_max_, Δρ_min_ (e Å^−3^)	0.51, −0.51

Computer programs: *APEX2* and *SAINT* (Bruker, 2010 ▸), *SHELXS97* and *SHELXTL* (Sheldrick, 2008 ▸) and *SHELXL2014* (Sheldrick, 2015 ▸).

## supplementary crystallographic information

### Crystal data

**Table:**

[Cu_2_Cl_4_(C_5_H_7_N_3_O_2_)_4_]·2C_2_H_3_N	*F*(000) = 932
*M**_r_* = 915.53	*D*_x_ = 1.678 Mg m^−^^3^
Monoclinic, *P*2_1_/*c*	Mo *K*α radiation, λ = 0.71073 Å
*a* = 13.9545 (8) Å	Cell parameters from 9452 reflections
*b* = 6.7004 (4) Å	θ = 2.9–30.6°
*c* = 19.5031 (11) Å	µ = 1.54 mm^−^^1^
β = 96.424 (1)°	*T* = 130 K
*V* = 1812.10 (18) Å^3^	Block, blue
*Z* = 2	0.35 × 0.17 × 0.10 mm

### Data collection

**Table:**

Bruker APEXII CCD diffractometer	5034 reflections with *I* > 2σ(*I*)
φ and ω scans	*R*_int_ = 0.033
Absorption correction: multi-scan (SADABS; Bruker, 2010)	θ_max_ = 30.6°, θ_min_ = 1.5°
*T*_min_ = 0.637, *T*_max_ = 0.746	*h* = −19→19
28822 measured reflections	*k* = −9→9
5564 independent reflections	*l* = −27→27

### Refinement

**Table:**

Refinement on *F*^2^	0 restraints
Least-squares matrix: full	Hydrogen site location: inferred from neighbouring sites
*R*[*F*^2^ > 2σ(*F*^2^)] = 0.024	H-atom parameters constrained
*wR*(*F*^2^) = 0.071	*w* = 1/[σ^2^(*F*_o_^2^) + (0.0371*P*)^2^] where *P* = (*F*_o_^2^ + 2*F*_c_^2^)/3
*S* = 1.26	(Δ/σ)_max_ = 0.033
5564 reflections	Δρ_max_ = 0.51 e Å^−^^3^
212 parameters	Δρ_min_ = −0.51 e Å^−^^3^

### Special details

**Table:**

Geometry. All esds (except the esd in the dihedral angle between two l.s. planes) are estimated using the full covariance matrix. The cell esds are taken into account individually in the estimation of esds in distances, angles and torsion angles; correlations between esds in cell parameters are only used when they are defined by crystal symmetry. An approximate (isotropic) treatment of cell esds is used for estimating esds involving l.s. planes.

### Fractional atomic coordinates and isotropic or equivalent isotropic displacement parameters (Å^2^)

**Table:**

	*x*	*y*	*z*	*U*_iso_*/*U*_eq_	
Cu	0.41957 (2)	0.46806 (2)	0.42320 (2)	0.01249 (5)	
Cl1	0.32308 (2)	0.45734 (5)	0.32075 (2)	0.02105 (7)	
Cl2	0.46436 (2)	0.25939 (4)	0.52033 (2)	0.01295 (6)	
O11	0.10021 (7)	0.39434 (18)	0.58607 (6)	0.0303 (2)	
O12	0.02709 (7)	0.66914 (16)	0.55203 (5)	0.0280 (2)	
O21	0.81805 (7)	0.13329 (16)	0.33794 (5)	0.0263 (2)	
O22	0.72749 (7)	−0.08394 (15)	0.38274 (6)	0.0276 (2)	
N11	0.09544 (8)	0.55179 (17)	0.55458 (6)	0.0209 (2)	
N12	0.18478 (7)	0.77681 (16)	0.48250 (5)	0.01611 (19)	
N13	0.30915 (7)	0.58276 (15)	0.46736 (5)	0.01353 (18)	
N21	0.74156 (7)	0.07990 (16)	0.35776 (5)	0.0175 (2)	
N22	0.65940 (7)	0.39673 (15)	0.31873 (5)	0.01363 (18)	
N23	0.53211 (7)	0.37776 (14)	0.37688 (5)	0.01264 (18)	
C11	0.17488 (8)	0.59978 (19)	0.51720 (6)	0.0164 (2)	
C12	0.25207 (9)	0.48130 (18)	0.50794 (6)	0.0152 (2)	
H12A	0.2639	0.3513	0.5264	0.018*	
C13	0.26757 (8)	0.76046 (17)	0.45264 (6)	0.0146 (2)	
C14	0.12067 (10)	0.9527 (2)	0.47787 (8)	0.0247 (3)	
H14A	0.1480	1.0572	0.4508	0.037*	
H14B	0.1145	1.0030	0.5244	0.037*	
H14C	0.0569	0.9147	0.4554	0.037*	
C15	0.30624 (9)	0.92050 (19)	0.41131 (7)	0.0189 (2)	
H15A	0.3555	0.8650	0.3847	0.028*	
H15B	0.3350	1.0252	0.4422	0.028*	
H15C	0.2538	0.9774	0.3797	0.028*	
C21	0.66334 (8)	0.21847 (17)	0.35462 (6)	0.0138 (2)	
C22	0.58485 (8)	0.20765 (17)	0.39040 (6)	0.0135 (2)	
H22A	0.5696	0.1011	0.4194	0.016*	
C23	0.57767 (8)	0.48769 (17)	0.33275 (6)	0.0131 (2)	
C24	0.72860 (10)	0.4744 (2)	0.27397 (7)	0.0218 (3)	
H24A	0.7509	0.3653	0.2463	0.033*	
H24B	0.7838	0.5330	0.3024	0.033*	
H24C	0.6973	0.5768	0.2433	0.033*	
C25	0.54213 (9)	0.68022 (18)	0.30190 (6)	0.0181 (2)	
H25A	0.4740	0.6973	0.3085	0.027*	
H25B	0.5491	0.6802	0.2525	0.027*	
H25C	0.5798	0.7902	0.3244	0.027*	

### Atomic displacement parameters (Å^2^)

**Table:**

	*U*^11^	*U*^22^	*U*^33^	*U*^12^	*U*^13^	*U*^23^
Cu	0.01138 (8)	0.01446 (8)	0.01188 (8)	0.00362 (5)	0.00239 (5)	−0.00060 (5)
Cl1	0.01728 (14)	0.02815 (16)	0.01670 (14)	0.00367 (11)	−0.00253 (11)	−0.00714 (11)
Cl2	0.01421 (12)	0.01058 (12)	0.01442 (12)	0.00041 (9)	0.00312 (9)	0.00093 (9)
O11	0.0229 (5)	0.0378 (6)	0.0318 (6)	0.0017 (4)	0.0102 (4)	0.0092 (5)
O12	0.0167 (4)	0.0344 (6)	0.0343 (6)	0.0063 (4)	0.0090 (4)	−0.0021 (4)
O21	0.0165 (4)	0.0332 (5)	0.0304 (5)	0.0058 (4)	0.0078 (4)	0.0035 (4)
O22	0.0259 (5)	0.0198 (4)	0.0379 (6)	0.0084 (4)	0.0066 (4)	0.0071 (4)
N11	0.0131 (5)	0.0292 (6)	0.0208 (5)	0.0021 (4)	0.0034 (4)	−0.0032 (4)
N12	0.0126 (4)	0.0188 (5)	0.0171 (5)	0.0049 (4)	0.0020 (4)	−0.0022 (4)
N13	0.0116 (4)	0.0151 (4)	0.0140 (4)	0.0015 (3)	0.0020 (3)	−0.0017 (3)
N21	0.0157 (5)	0.0211 (5)	0.0157 (5)	0.0038 (4)	0.0018 (4)	−0.0016 (4)
N22	0.0139 (4)	0.0151 (4)	0.0122 (4)	0.0009 (4)	0.0029 (3)	0.0007 (3)
N23	0.0135 (4)	0.0125 (4)	0.0122 (4)	0.0023 (3)	0.0026 (3)	0.0003 (3)
C11	0.0131 (5)	0.0211 (6)	0.0155 (5)	0.0017 (4)	0.0035 (4)	−0.0016 (4)
C12	0.0132 (5)	0.0173 (5)	0.0153 (5)	0.0001 (4)	0.0022 (4)	−0.0009 (4)
C13	0.0116 (5)	0.0171 (5)	0.0148 (5)	0.0015 (4)	0.0001 (4)	−0.0034 (4)
C14	0.0214 (6)	0.0248 (7)	0.0287 (7)	0.0140 (5)	0.0061 (5)	0.0000 (5)
C15	0.0190 (6)	0.0162 (5)	0.0217 (6)	0.0024 (4)	0.0030 (5)	0.0019 (4)
C21	0.0139 (5)	0.0143 (5)	0.0135 (5)	0.0031 (4)	0.0020 (4)	−0.0008 (4)
C22	0.0154 (5)	0.0122 (5)	0.0130 (5)	0.0023 (4)	0.0025 (4)	0.0005 (4)
C23	0.0142 (5)	0.0133 (5)	0.0118 (5)	0.0007 (4)	0.0015 (4)	−0.0009 (4)
C24	0.0190 (6)	0.0278 (7)	0.0202 (6)	0.0008 (5)	0.0087 (5)	0.0068 (5)
C25	0.0207 (6)	0.0158 (5)	0.0186 (6)	0.0031 (4)	0.0049 (4)	0.0047 (4)

### Geometric parameters (Å, º)

**Table:**

Cu—N23	1.9914 (9)	N22—C24	1.4678 (16)
Cu—N13	2.0009 (10)	N23—C23	1.3455 (15)
Cu—Cl1	2.2822 (3)	N23—C22	1.3663 (14)
Cu—Cl2	2.3811 (3)	C11—C12	1.3662 (16)
Cu—Cl2^i^	2.6024 (3)	C12—H12A	0.9500
Cl2—Cu^i^	2.6024 (3)	C13—C15	1.4792 (17)
O11—N11	1.2188 (16)	C14—H14A	0.9800
O12—N11	1.2329 (14)	C14—H14B	0.9800
O12—N11^ii^	2.9399 (15)	C14—H14C	0.9800
O21—N21	1.2285 (14)	C15—H15A	0.9800
O22—N21	1.2258 (15)	C15—H15B	0.9800
N11—C11	1.4299 (16)	C15—H15C	0.9800
N11—O12^ii^	2.9399 (15)	C21—C22	1.3649 (16)
N12—C13	1.3552 (15)	C22—H22A	0.9500
N12—C11	1.3802 (16)	C23—C25	1.4850 (16)
N12—C14	1.4764 (15)	C24—H24A	0.9800
N13—C13	1.3414 (15)	C24—H24B	0.9800
N13—C12	1.3653 (15)	C24—H24C	0.9800
N21—C21	1.4291 (15)	C25—H25A	0.9800
N22—C23	1.3478 (15)	C25—H25B	0.9800
N22—C21	1.3823 (15)	C25—H25C	0.9800
			
N23—Cu—N13	175.06 (4)	N13—C12—H12A	126.1
N23—Cu—Cl1	90.65 (3)	N13—C13—N12	110.42 (11)
N13—Cu—Cl1	88.95 (3)	N13—C13—C15	125.78 (11)
N23—Cu—Cl2	91.85 (3)	N12—C13—C15	123.78 (11)
N13—Cu—Cl2	91.68 (3)	N12—C14—H14A	109.5
Cl1—Cu—Cl2	139.002 (12)	N12—C14—H14B	109.5
N23—Cu—Cl2^i^	85.42 (3)	H14A—C14—H14B	109.5
N13—Cu—Cl2^i^	91.21 (3)	N12—C14—H14C	109.5
Cl1—Cu—Cl2^i^	132.139 (12)	H14A—C14—H14C	109.5
Cl2—Cu—Cl2^i^	88.842 (10)	H14B—C14—H14C	109.5
Cu—Cl2—Cu^i^	91.158 (10)	C13—C15—H15A	109.5
N11—O12—N11^ii^	95.37 (8)	C13—C15—H15B	109.5
O11—N11—O12	124.80 (11)	H15A—C15—H15B	109.5
O11—N11—C11	116.73 (10)	C13—C15—H15C	109.5
O12—N11—C11	118.46 (11)	H15A—C15—H15C	109.5
O11—N11—O12^ii^	85.14 (8)	H15B—C15—H15C	109.5
O12—N11—O12^ii^	84.63 (8)	C22—C21—N22	108.42 (10)
C11—N11—O12^ii^	100.10 (7)	C22—C21—N21	126.61 (11)
C13—N12—C11	106.15 (10)	N22—C21—N21	124.75 (10)
C13—N12—C14	125.32 (11)	C21—C22—N23	107.62 (10)
C11—N12—C14	128.53 (10)	C21—C22—H22A	126.2
C13—N13—C12	107.43 (10)	N23—C22—H22A	126.2
C13—N13—Cu	125.75 (8)	N23—C23—N22	110.69 (10)
C12—N13—Cu	125.84 (8)	N23—C23—C25	125.06 (10)
O21—N21—O22	124.71 (11)	N22—C23—C25	124.23 (11)
O21—N21—C21	118.93 (11)	N22—C24—H24A	109.5
O22—N21—C21	116.32 (10)	N22—C24—H24B	109.5
C23—N22—C21	105.94 (9)	H24A—C24—H24B	109.5
C23—N22—C24	126.03 (10)	N22—C24—H24C	109.5
C21—N22—C24	128.03 (10)	H24A—C24—H24C	109.5
C23—N23—C22	107.29 (9)	H24B—C24—H24C	109.5
C23—N23—Cu	125.24 (8)	C23—C25—H25A	109.5
C22—N23—Cu	126.94 (8)	C23—C25—H25B	109.5
C12—C11—N12	108.11 (10)	H25A—C25—H25B	109.5
C12—C11—N11	127.19 (12)	C23—C25—H25C	109.5
N12—C11—N11	124.69 (10)	H25A—C25—H25C	109.5
C11—C12—N13	107.88 (11)	H25B—C25—H25C	109.5
C11—C12—H12A	126.1		
			
N11^ii^—O12—N11—O11	−80.27 (13)	C11—N12—C13—C15	−178.76 (11)
N11^ii^—O12—N11—C11	98.59 (11)	C14—N12—C13—C15	0.78 (19)
N11^ii^—O12—N11—O12^ii^	0.0	C23—N22—C21—C22	−1.06 (13)
C13—N12—C11—C12	0.40 (13)	C24—N22—C21—C22	179.19 (11)
C14—N12—C11—C12	−179.12 (12)	C23—N22—C21—N21	−175.98 (11)
C13—N12—C11—N11	−178.29 (11)	C24—N22—C21—N21	4.28 (19)
C14—N12—C11—N11	2.2 (2)	O21—N21—C21—C22	−160.72 (12)
O11—N11—C11—C12	6.01 (19)	O22—N21—C21—C22	16.86 (18)
O12—N11—C11—C12	−172.94 (12)	O21—N21—C21—N22	13.27 (17)
O12^ii^—N11—C11—C12	−83.60 (13)	O22—N21—C21—N22	−169.15 (11)
O11—N11—C11—N12	−175.55 (12)	N22—C21—C22—N23	0.11 (13)
O12—N11—C11—N12	5.50 (18)	N21—C21—C22—N23	174.90 (11)
O12^ii^—N11—C11—N12	94.84 (12)	C23—N23—C22—C21	0.90 (13)
N12—C11—C12—N13	−0.50 (13)	Cu—N23—C22—C21	−171.07 (8)
N11—C11—C12—N13	178.15 (11)	C22—N23—C23—N22	−1.62 (13)
C13—N13—C12—C11	0.40 (13)	Cu—N23—C23—N22	170.52 (7)
Cu—N13—C12—C11	−168.81 (8)	C22—N23—C23—C25	176.82 (11)
C12—N13—C13—N12	−0.15 (13)	Cu—N23—C23—C25	−11.04 (16)
Cu—N13—C13—N12	169.07 (8)	C21—N22—C23—N23	1.66 (13)
C12—N13—C13—C15	178.42 (11)	C24—N22—C23—N23	−178.59 (11)
Cu—N13—C13—C15	−12.36 (17)	C21—N22—C23—C25	−176.79 (11)
C11—N12—C13—N13	−0.16 (13)	C24—N22—C23—C25	2.96 (19)
C14—N12—C13—N13	179.38 (11)		

Symmetry codes: (i) −*x*+1, −*y*+1, −*z*+1; (ii) −*x*, −*y*+1, −*z*+1.

### Hydrogen-bond geometry (Å, º)

**Table:**

*D*—H···*A*	*D*—H	H···*A*	*D*···*A*	*D*—H···*A*
C25—H25*C*···O22^iii^	0.98	2.39	3.2804 (17)	150
C15—H15*B*···Cl2^iii^	0.98	2.73	3.6737 (13)	163
C12—H12*A*···O22^iv^	0.95	2.51	3.4029 (16)	156
C22—H22*A*···Cl2^iv^	0.95	2.75	3.6828 (12)	167
C24—H24*C*···Cl1^v^	0.98	2.84	3.7555 (13)	156

Symmetry codes: (iii) *x*, *y*+1, *z*; (iv) −*x*+1, −*y*, −*z*+1; (v) −*x*+1, *y*+1/2, −*z*+1/2.
